# Supplementary material for: Cryo-Electron Microscopy of BfpB Reveals a Type IVb Secretin Multimer Adapted to Accommodate the Exceptionally Wide Bundle-Forming Pilus
Source: Pathogens. 2025 May 13;14(5):471. doi: 10.3390/pathogens14050471 (PMC12114550; doi:10.3390/pathogens14050471)
Supplement: Supplementary file 1 [file pathogens-14-00471-s001.zip › pathogens-3475234-supplementary.pdf]

A

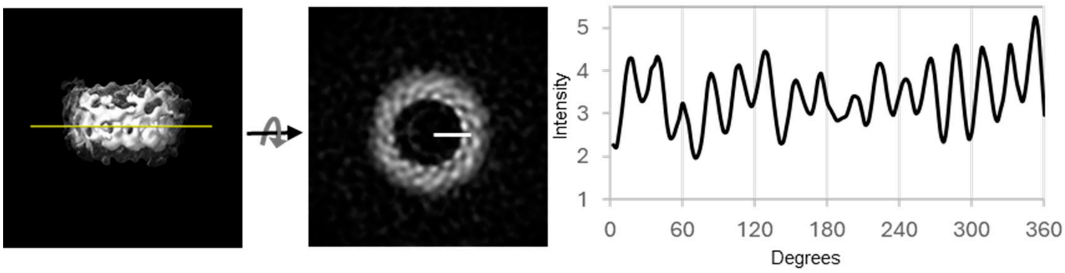

B

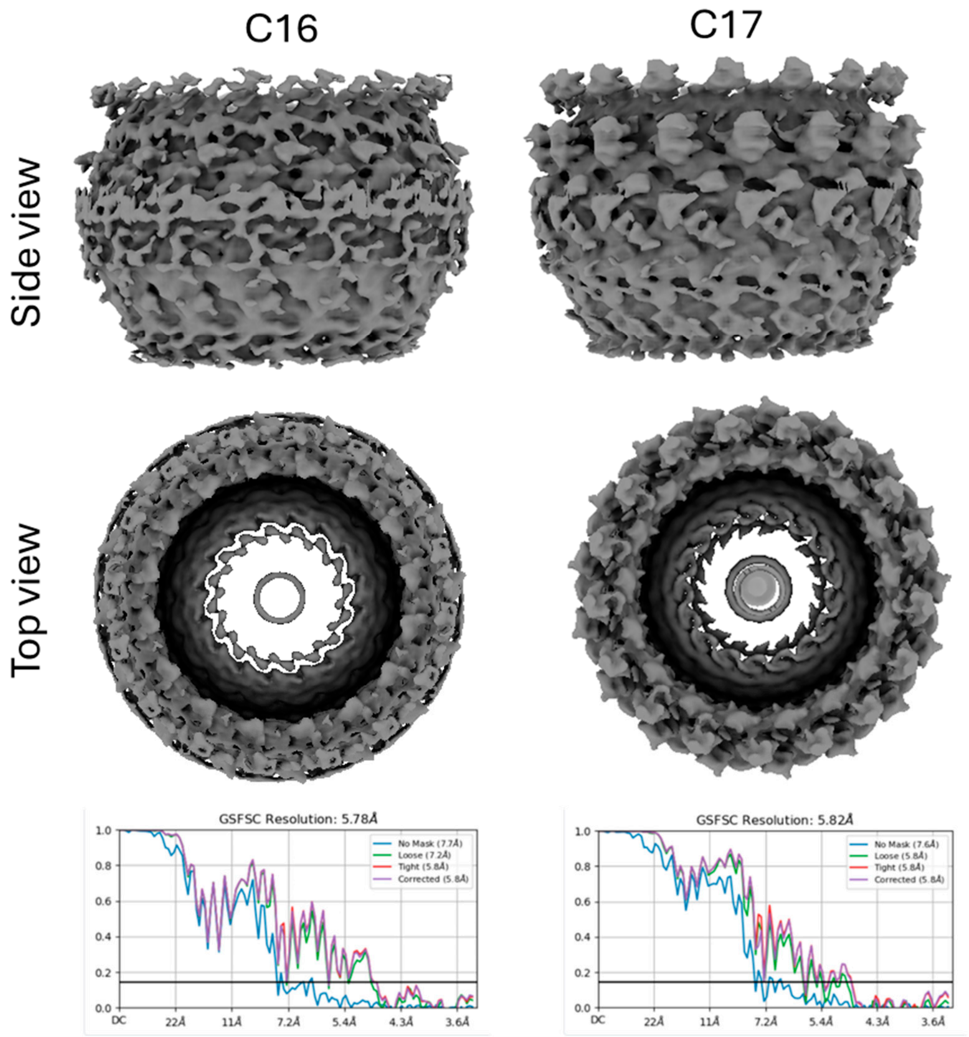

Supplementary Figure S1: Analysis of C16/ C17 symmetry ambiguity.

Ab-initio 3D classification without symmetry imposed resulted in five classes: three with recognizable structural features and two containing amorphous particles. One of the classes with recognizable features displayed ambiguous symmetry between C16 and C17. **(A)** A polar coordinate plot from a 2D slice (yellow line) of the 3D volume of the ambiguous class shows variable separation of the intensity peaks, with most of them conforming to C17 symmetry. The mesh displays the isosurface (Fig. 3B) at a lower threshold. **(B)** Particles from the ambiguous class were refined separately with imposed C16 and C17 symmetries. The side and top views of the resulting maps are shown, highlighting structural differences and less resolved features when using C16 symmetry. The bottom panel shows the GSFSC curves for both models, with a pronounced local minimum at ~15 Å for the C16-symmetrized map, indicating potential misalignment or symmetry mismatch.

A

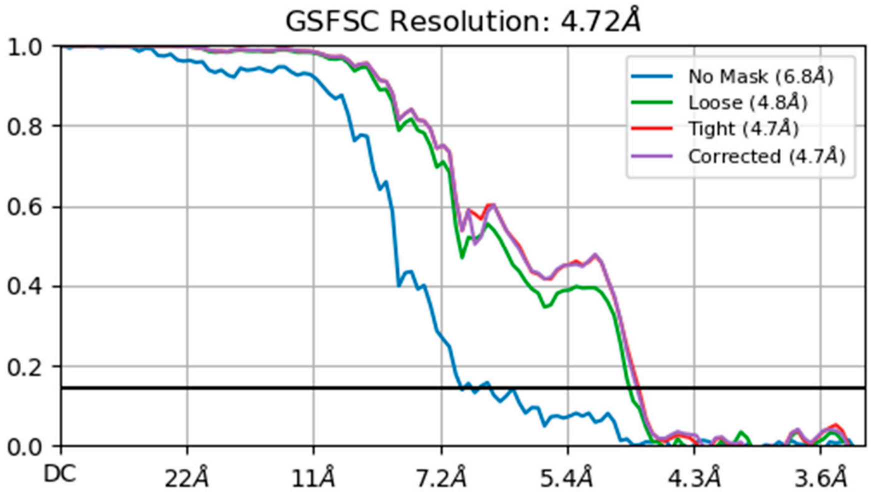

B

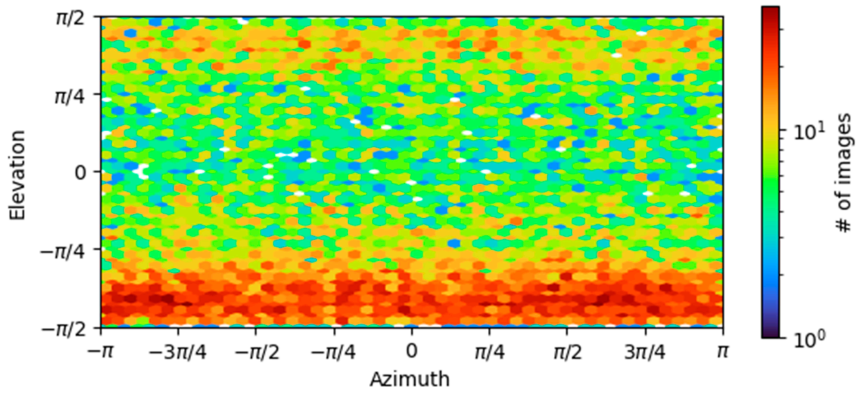

Supplementary Figure S2: (A) Gold-standard Fourier shell correlation (GSFSC) curve for the final 3D reconstruction of BfpB, indicating an overall resolution of 4.7 Å at the 0.143 cutoff. **(B)** Distribution of particle orientations used in the final reconstruction, showing non-uniform angular coverage.

```
InvG_S-domain     524 PDASESV-NNILK--QSGAWSGDDKLQKWVRVY 524
GspD_S-domain     571 IIRDDDVYRSLSK--EKYTRYRQEQQQRIDGKS 599
BfpB_N-helix       19 --SGNGFYKDNLGviDKNILHADTSLLKSK--- 46
```

Supplementary Figure S3. Multiple sequence alignment between the S-domains of InvG and GspD and the amino-terminal helix of BfpB. The alignment was done using NIH-COBALT tool (Papadopoulos & Agarwala, 2007) with a setting of Gap penalties (-11, -1) and end gap penalties (-5, -1).

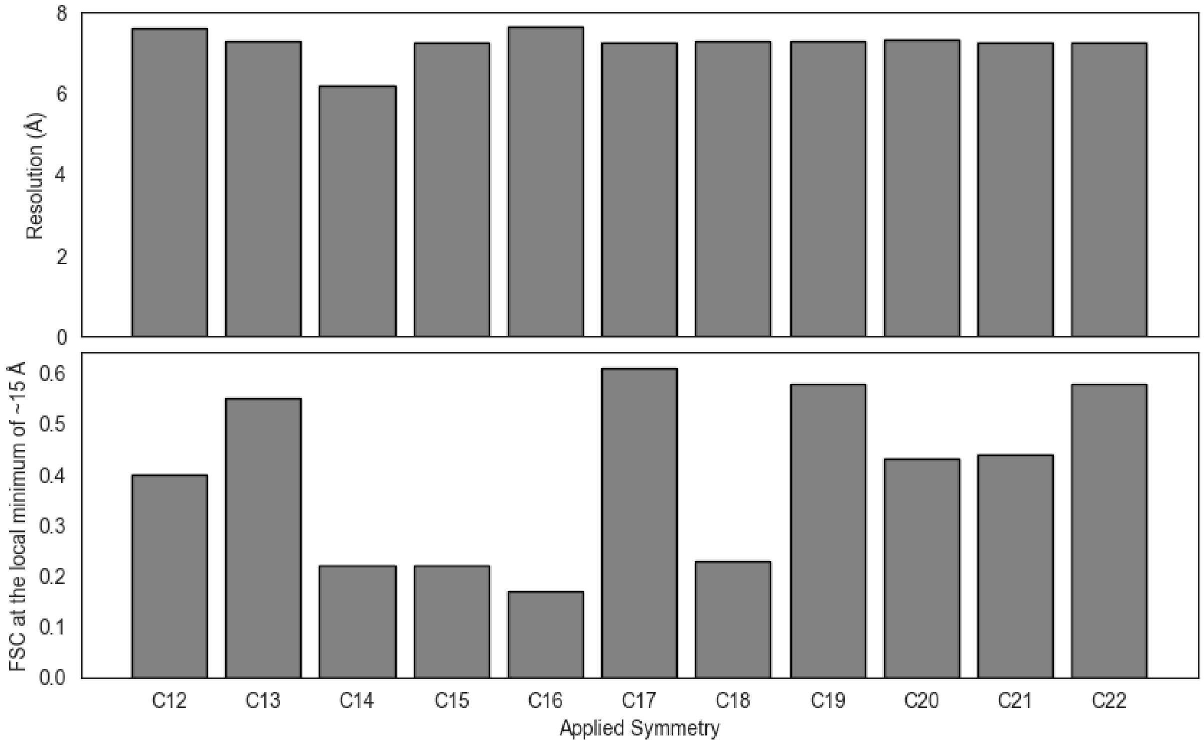

Supplementary Figure S4: Supplementary Figure 4: Symmetry assessment of BfpB.

Particles from the final C17 model were processed *de novo,* performing 3D reconstruction and refinement with symmetries ranging from C12 to C22. The resulting resolution values at GSFSC 0.143 cutoff were relatively homogeneous across symmetries, with the

exception of C14, which was slightly higher. Additionally, the GSFSC curves showed a pronounced local minimum at ~15 Å for reconstructions with C12, C14, C15, C16, C18, C20, and C21 symmetries, which suggests misalignment or symmetry mismatch. In contrast, the GSFSC curves in the case of C13, C17, C19 & C22 had a less pronounced local minimum at ~15Å. Of these, C17 showed the highest signal at the 15 Å mark and the most detailed map features, as well as agreement with the morphological features visible in the C1 Ab-initio classification data (Fig. 3B). Therefore, subsequent refinement was performed using C17 symmetry, which led to a final resolution of 4.7 Å.

Table S1: Data collection, image processing, and model statistics

**Data Acquisition**	**BfpB**
Microscope/Detector	Krios/K3
Voltage (kV)	300
Magnification	54,000
Spherical Aberration (mm)	2.7
Data collection mode	Counting
Pixel Size (Å) (super-resolution)	1.68
Defocus range (µm)	-1. to -2.25
Total electron dose (e/Å2) (Number of frames)	50
Total number of movies	9,248
**Image Processing**	
Total number of particles picked	255,000
Particles after 2D classification	33,096
Particles used for 3D refinement	25,675
Resolution (Å)	4.7

EMDB ID	EMD-70296

References:

Papadopoulos, J. S., & Agarwala, R. (2007). COBALT: Constraint-based alignment tool for multiple protein sequences. *Bioinformatics*, *23*(9), 1073–1079. https://doi.org/10.1093/bioinformatics/btm076
